# Use of advanced recombinant lines to study the impact and potential of mutations affecting starch synthesis in barley^☆^

**DOI:** 10.1016/j.jcs.2013.12.012

**Published:** 2014-03

**Authors:** Thomas P. Howard, Brendan Fahy, Fiona Leigh, Phil Howell, Wayne Powell, Andy Greenland, Kay Trafford, Alison M. Smith

**Affiliations:** a Department of Metabolic Biology, John Innes Centre, Norwich Research Park, Norwich NR4 7UH, United Kingdom; b National Institute of Agricultural Botany, Huntingdon Road, Cambridge CB3 OLE, United Kingdom

**Keywords:** Barley mutant, Barley starch, Starch properties

## Abstract

The effects on barley starch and grain properties of four starch synthesis mutations were studied during the introgression of the mutations from diverse backgrounds into an elite variety. The *lys5f* (ADPglucose transporter), *wax* (granule-bound starch synthase), *isa1* (debranching enzyme isoamylase 1) and *sex6* (starch synthase IIa) mutations were introgressed into NFC Tipple to give mutant and wild-type BC_2_F_4_ families with different genomic contributions of the donor parent. Comparison of starch and grain properties between the donor parents, the BC_2_F_4_ families and NFC Tipple allowed the effects of the mutations to be distinguished from genetic background effects. The *wax* and *sex6* mutations had marked effects on starch properties regardless of genetic background. The *sex6* mutation conditioned low grain weight and starch content, but the *wax* mutation did not. The *lys5* mutation conditioned low grain weight and starch content, but exceptionally high β-glucan contents. The *isa*1 mutation promotes synthesis of soluble α-glucan (phytoglycogen). Its introgression into NFC Tipple increased grain weight and total α-glucan content relative to the donor parent, but reduced the ratio of phytoglycogen to starch. This study shows that introgression of mutations into a common, commercial background provides new insights that could not be gained from the donor parent.

## Highlights

• Four barley starch synthesis mutations were introgressed from diverse backgrounds into an elite variety.
• *wax* and *sex6* mutations had marked effects on starch properties regardless of genetic background.
• *sex6* and *lys5* mutations reduced grain weight, but the *lys5* mutation led to very high β-glucan contents.
• Introgression reduced the phytoglycogen (soluble α-glucan) to starch ratio in *isa1* mutants.

## 1 Introduction

The aim of this work was to characterise variation for starch properties in barley, and to provide new barley germplasm for evaluation of the commercial potential of this variation. Starch is the main component of barley grains, and the substrate for production of alcohol in the barley-based drinks industries.

The commercial application and value of a particular starch depends on its physical properties, including gelatinisation profile, gel and paste properties, and susceptibility to hydrolysis. Much starch used by industry is chemically, physically or enzymatically modified to tailor its properties to specific end uses (Huber and BeMiller, 2009). Industry also exploits naturally-occurring variation for starch properties within and between starch crops. For example, starch from the *waxy* mutant of maize gelatinises to form clear pastes with specific applications in the food industry, and “high-amylose” maize starch (from the *amylose-extender* mutant) is particularly valued for its film-forming properties (Jobling, 2004). Naturally-occurring variation in starch properties also influences the quality of foods made from starch crops. For example, the *waxy* mutant of rice has eating properties that are particularly prized in East Asia, and flour from high-amylose maize gives baked products with low glycaemic indices because of the resistance of the starch to digestion in the gut (Behall and Hallfrisch, 2002; Nugent, 2005).

Some variation for starch properties exists within commercial barley varieties. A variety containing starch with a high amylose content (Bird et al., 2004; Morell et al., 2003; Topping et al., 2003) is marketed as a healthy breakfast cereal in Australia, and varieties with different kinds of resistant starch (starch that escapes digestion in the upper gut and enters the large bowel) are under development (Regina et al., 2010). Waxy barley is cultivated in North America because its flour confers freeze-thaw and antistaling properties to processed food. Waxy barley is also sold as a health food because the grains have a high content of beta-glucan, an important component of dietary fibre (Baik and Ullrich, 2008; Ullrich et al., 1986).

A wide range of variation for starch structure and properties exists within barley germplasm collections, but is not currently commercially exploited. Many different mutants with altered granule composition and/or morphology have been reported (e.g. DeHaas et al., 1983; Oscarsson et al., 1997; Patron et al., 2004; Tester et al., 1993). The introduction of novel variation into high-yielding commercial cultivars could potentially open new markets for barley, and generate new products. However, progress in evaluating starch variation in germplasm collections for this purpose is slow because the variation is scattered through many different genetic backgrounds. It is difficult to determine the extent to which a given starch property is modulated by genetic background, or whether altered starch properties may have negative impacts on desirable traits including yield.

To overcome these problems, potentially important starch traits could be transferred to a common, elite genetic background. To this end, we used marker-assisted selection to transfer four different mutations with major effects on starch properties from diverse accessions (donor parents) into the UK elite variety NFC Tipple (Howell et al., 2014). Here we compare properties of grain from the mutant and wild-type parental lines with those of grain from a series of recombinant BC_2_F_4_ families, each with different proportions of donor genotype. The results allow meaningful evaluation of the effects of different mutations on starch and grain properties, and the impact of genetic background on these properties.

The four mutations condition different starch properties and come from three very different genetic backgrounds. Our choices thus maximise both the research and exploitation value of the introgression lines from a starch perspective, and the amount of other genetic diversity introduced into NFC Tipple for future studies. Two of the mutations (*sex6* and *wax*) have already been exploited commercially in other genetic backgrounds, providing potentially useful comparisons of genetic background effects. The other two mutations bring about starch and grain properties that are not found in existing commercial cultivars of barley. The genes affected were as follows.

The gene at the *sex6* locus encodes starch synthase IIa (SSIIa; Morell et al., 2003). Starch from the mutant is highly resistant to digestion, and grains have health benefits including low glycaemic indices and high soluble fibre (e.g. Bird et al., 2004; Rahman et al., 2007; Topping et al., 2003). The gene at the *lys5* locus encodes an ADPglucose transporter, responsible for transferring ADPglucose synthesised in the cytosol into the amyloplast where it is the substrate for starch synthesis (Patron et al., 2004). The gene at the *wax* locus encodes granule-bound starch synthase I (GBSSI). The mutation conditions the loss of amylose from endosperm starch (Ishikawa et al., 1995; Patron et al., 2002). Amylose-free and low-amylose starches have food applications because of their low gelatinisation temperatures, high peak and low final viscosities, and high freeze-thaw stability (Baik and Ullrich, 2008). The gene at the *isa1* locus encodes isoamylase 1, a starch debranching enzyme. Barley *isa1* mutants (Risø17 and Notch2) have low starch contents, but high contents of a soluble α-glucan, phytoglycogen (Burton et al., 2002).

## 2 Materials and methods

### 2.1 Plant material

Development of recombinant pre-breeding lines from crosses between NFC Tipple and four barley accessions carrying mutations affecting starch synthesis was described in Howell et al. (2014). Mutant accessions were: Risø13 (*lys5f*), Yon M Kei (*wax*), Notch2 (*isa1*), sex6 (*sex6*). Risø13 was obtained from the John Innes Centre Germplasm Resources Unit (http://www.jic.ac.uk/germplasm/). Yon M Kei was obtained from Dr. Naoyuki Ishikawa (Tochigi Agricultural Experiment Station, Tochigi, Japan). Notch2 (NP113; PI 392251) and sex6 (GSHO 2476) were obtained from the National Small Grains Collection, Idaho, USA.

Seed of BC_2_F_4_ recombinants and all parental lines was sown in the field in 2 m × 6 row plots at the National Institute of Agricultural Botany (NIAB TAG, Cambridge, UK) in 2010. BC_2_F_5_ grain was harvested with a plot combine as the bulk of four BC_2_F_4_ plots, with each bulk representing a different original BC_2_F_2_ individual.

The material analysed in this study is listed in Table 1. High, medium and low donor classification was based upon the genome-wide DArT genotypes of the BC_1_ and BC_2_ generations (Howell et al., 2014). For each mutation, the low donor line and wild-type control represent a pair of full-sib near-isogenic lines, selected as contrasting BC_2_F_2_ homozygotes from within a segregating family. The *lys5* low donor bulk was generated by pooling equal quantities of grain from three low donor lines; similar low donor bulks were generated for *wax* from five low donor lines and for *isa1* and *sex6* from two low donor lines each.

### 2.2 Starch extraction and purification

Methods were based on those of South and Morrison (1990) and Sulaiman and Morrison (1990). Samples of grain (25 g) were milled to flour in a Perten Laboratory Mill (LM3100, www.perten.com), mixed with 500 ml water at 4 °C for 1 h then filtered through two layers of muslin and one layer of Miracloth. The filtrate was centrifuged for 5 min at 1300 g and the supernatant discarded. The pellet was suspended in 12 ml water. Samples of 0.75 ml were overlaid on 8 ml 80% caesium chloride in 15 ml tubes and centrifuged for 10 min at 2400 g. The top layer was gently mixed, and the tubes re-centrifuged. The supernatant was discarded and the starch pellet was washed once with 2% (w/v, aq.) SDS, three times with water and twice with acetone then air-dried and stored at −20 °C.

### 2.3 Starch, phytoglycogen and β-glucan content

For most measurements, samples of 2 mg flour were incubated in 1 ml 80% ethanol for 20 min at room temperature, centrifuged at 14,000 *g*, and the supernatant discarded. This process was repeated, then the pellet was suspended in 1 ml water, autoclaved to solubilise starch, and assayed by digestion to glucose followed by enzymatic assay of glucose as described by Burton et al. (2002). For measurements shown in Fig. 2B, granular (water-insoluble) starch and water-soluble phytoglycogen were assayed separately, as described by Burton et al. (2002). Flour was separated into water insoluble and water-soluble material. Phytoglycogen was precipitated from water-soluble material with aqueous methanol/KCl. β-Glucan was assayed with a mixed-linkage β-glucan assay kit (Megazyme), according to the manufacturer's instructions except that the system was scaled down for samples of 15–20 mg flour and glucose was assayed according to Smith and Zeeman (2006).

### 2.4 Differential Scanning Calorimetry (DSC)

Analysis was performed as a technical service by Campden BRI (http://www.campdenbri.co.uk/), using a Perkin Elmer Pyris 1 DSC (www.perkinelmer.co.uk). Samples consisted of 10 mg starch, 20 mg water. Heating was from 25 °C to 100 °C at 10 °C min^−1^.

### 2.5 Rapid Visco-Analysis

RVA was carried out by Campden BRI (see Section 2.4) using the Newport standard profile 1 (Newport Scientific, Warriewood, NSW, Australia) on suspensions of 2 g starch (based on a measured moisture content of 12%) in 23.0 g water.

### 2.6 Starch swelling power and freeze-thaw stability

For swelling power, samples of 10 mg starch were suspended in 1 ml water and incubated at 80 °C, 750 rpm in a thermomixer for 20 min then cooled and centrifuged at 1500 g for 5 min. The supernatant was removed and the weight of the starch pellet determined. Swelling power was calculated as the fractional increase in weight of starch over the process. For freeze-thaw stability, samples of 25 mg starch were suspended in 1 ml water, heated as above, cooled to room temperature then frozen at −80 °C overnight, thawed for 1 h, and centrifuged at 8000 g for 15 min. The supernatant was removed and the freeze-thaw cycle repeated. Freeze-thaw stability was calculated as the percentage weight loss of the gelatinised starch after five freeze-thaw cycles.

### 2.7 Grain weight and size

Measurements were made with a Marvin 4.0 seed analyser/counter (GTA Sensorik, Neubrandenburg, Germany) on between 230 and 370 grains per line.

## 3 Results

Grain and starch properties were analysed on samples harvested from NFC Tipple, the original mutant accessions (referred to as donor parents), and BC_2_F_4_ recombinant lines developed from crosses between NFC Tipple and the four donor parents. Three categories of mutant families were selected: carrying relatively high, low or medium levels of donor genotype across their genomes (Table 1). These are referred to as high, low and medium donor families, respectively, low-donor families being those with the highest representation of the recurrent parent NFC Tipple in their genomes. Some measurements were carried out on grain samples from the low donor bulks, whilst for others, an individual family from each category was analysed.

### 3.1 Grain characteristics

For all of the physical characteristics examined, grain from wild-type BC_2_F_4_ families closely resembled grain from NFC Tipple (values within 6% of NFC Tipple), whereas mutant families displayed a much wider range of variation.

**Fig. 1:**
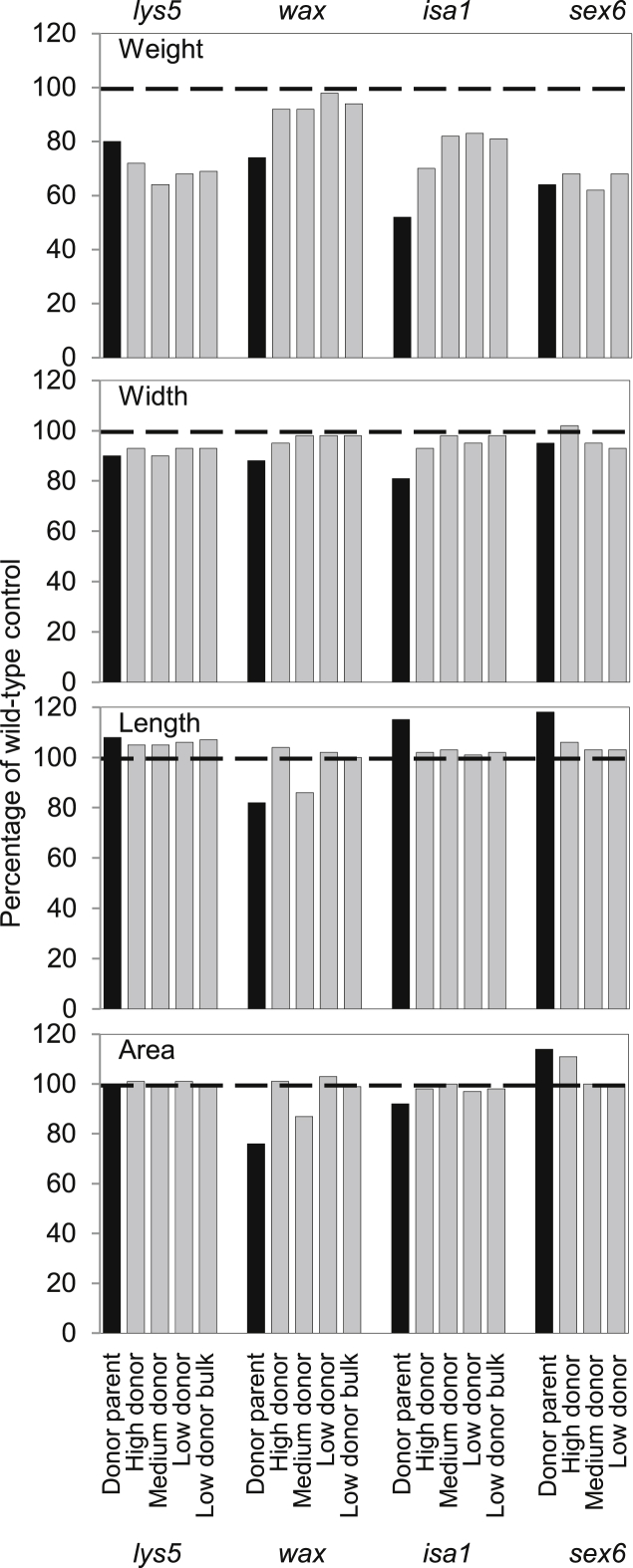
Grain weight, width, length and area. Measurements on grain from donor parents (black bars) and mutant BC_2_F_4_ families (see Supplementary Table 1) are expressed as a percentage of the wild-type control value (100%, dashed line). Donor parents are the original mutant accessions. High, medium and low donor lines carry high, low or medium levels of donor genotype across their genomes, respectively. Wild-type controls are full sibs of the low donor line for each mutation. Low donor bulks were created by pooling equal quantities of grains from two to five low donor lines (see Table 1). Wild-type control values as a percentage of the NFC Tipple value were as follows. Weight: *lys5* 94, *wax* 98, *isa1* 100, *sex6* 96. Width: *lys5* 98, *wax* 98, *isa1* 100, *sex6* 98. Length: *lys5* 99, *wax* 97, *isa1* 99, *sex6* 99. Area: *lys5* 98, *wax* 94, *isa1* 101, *sex6* 97.

Introgression into NFC Tipple confirmed that the four mutations have markedly different impacts on grain weight (Fig. 1, Supplementary Table 1). The *wax* mutation had little impact on grain weight. Although the grain weight of the donor parent was 25% less than that of the wild-type control, grain weights of all of the *wax* mutant families were within 8% of that of the wild-type control. The grain weight of the donor parent for the *isa1* mutation was only 50% of that of the wild-type control. Grain weights of the *isa* mutant families were all greater than that of the donor parent, ranging from 70% (high donor family) to 83% (low donor family) of the wild-type control. In contrast, for *sex6* and *lys5* mutant families, grain weight was comparable with that of the donor parent and only 62–72% of the wild-type control regardless of the donor contribution to the genetic background (Fig. 1).

None of the mutations influenced the length, width or area of the grain. Although donor parents were in several cases markedly different from the wild-type control, grains from low-donor families almost all closely resembled those of the wild-type control (within 7% of wild-type control values). One exception was the *wax* medium donor BC_2_F_4_ family, which had shorter grains of smaller area than either the high or low donor families, and was comparable in these respects with the donor parent. This difference is due to segregation for the hulless characteristic: grains of the donor parent and the medium donor family are hulless whereas NFC Tipple and the other families tested are hulled.

### 3.2 Starch, phytoglycogen and β-glucan content

**Fig. 2:**
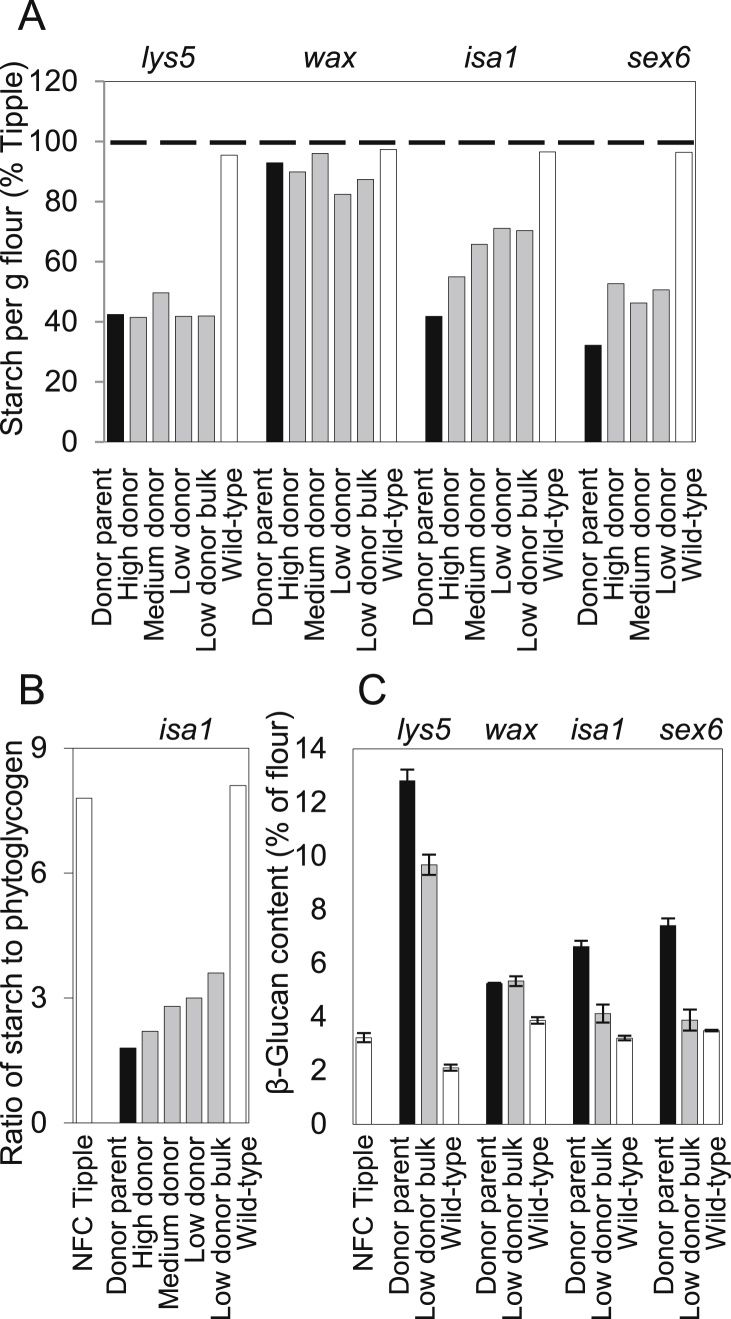
Starch and glucan contents. (A) Starch content per g flour from grain of donor parents (black bars), mutant BC_2_F_4_ families (grey bars) and wild-type controls (white bars), expressed as percentages of the value for NFC Tipple (100%, dashed line). Primary measurements are provided in Supplementary Table 2. (B) Ratio of starch to phytoglycogen in flour from grain of NFC Tipple, the *isa1* donor parent and *isa1* BC_2_F_4_ families. (C) β-Glucan content as a percentage of flour from grain of donor parents (black bars), mutant BC_2_F_4_ families (grey bars), and NFC Tipple and wild-type controls (white bars). Primary measurements are provided in Supplementary Table 3. Nomenclature of the material is explained in Fig. 1.

Introgression of the mutations into NFC Tipple affected starch content in approximately the same way as it affected grain weight (Fig. 2A, Supplementary Table 2). Starch content per g flour was comparable with that of NFC Tipple in the wax mutant donor and in BC_2_F_4_ families carrying this mutation. BC_2_F_4_ families carrying the *lys5* mutation all had very low starch contents, comparable with that of the donor parent (about 40% of NFC Tipple). Starch content in grains of BC_2_F_4_ families carrying the *sex6* mutation was about one third higher than in the donor parent, but still only half of NFC Tipple values.

Grain of *isa1* mutants contains a large proportion of its carbohydrate as a soluble, branched α-glucan, phytoglycogen, rather than as starch (Burton et al., 2002). Separate measurements of starch (water-insoluble α-glucan) and phytoglycogen (water-soluble α-glucan) revealed that, as expected, grain from the *isa* donor parent had a much lower starch content than NFC Tipple and the wild-type control, and its phytoglycogen was almost 60% of its starch content (Fig. 2B, Supplementary Table 3). Grain from mutant BC_2_F_4_ families had higher contents of starch and of starch plus phytoglycogen than the donor parent, the highest contents being in the lines with the lowest donor contribution to the genetic background. The increase in starch content in the mutant families was not accompanied by an increase in phytoglycogen. Grain from the low-donor mutant family had the same phytoglycogen content as the donor parent (124 ± 19 mg g^−1^ flour and 116 ± 34 mg g^−1^ respectively, mean ± SE of values from three separately-prepared batches of flour), but twice the starch to phytoglycogen ratio (Fig. 2B).

Barley grains are rich in β-glucan, an important component of dietary fibre (Havrlentová and Kraic, 2006). Grain of all four donor parents had higher β-glucan contents (as % flour) than NFC Tipple (Fig. 2C). In the *lys5* donor, β-glucan content was four times higher than in NFC Tipple. Although β-glucan content was lower in bulked, low-donor *lys5* mutant families, it was still three times higher than in NFC Tipple. β-glucan contents of *wax* mutant families were about 40% higher than that of Tipple and comparable with that of the donor parent. In contrast, the bulked, low-donor mutant families for *isa1* and *sex6* had β-glucan contents similar to that of NFC Tipple rather than the donor parent.

### 3.3 Differential Scanning Calorimetry and Rapid Visco Analysis

**Fig. 3:**
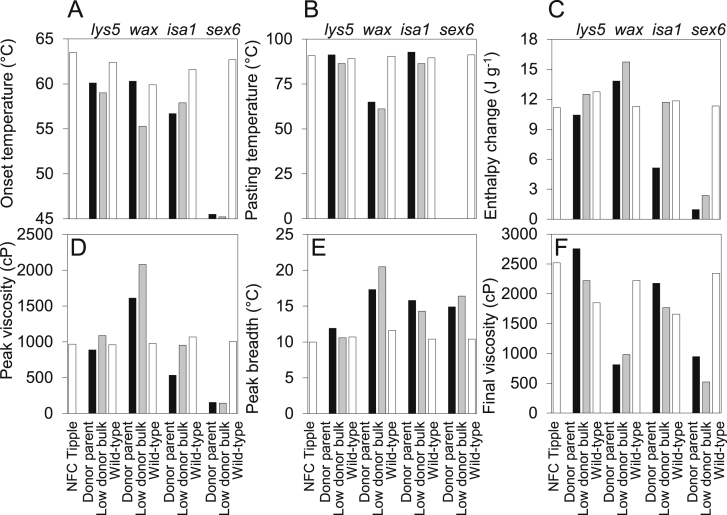
Gelatinisation and pasting characteristics of starches from grain of donor parents (black bars), mutant BC_2_F_4_ families (grey bars), and NFC Tipple and wild-type controls (white bars). Values are means of measurements on duplicate samples from the same batch of starch. Differences between duplicates were <5%. (A) Onset temperature for the DSC gelatinisation endotherm. (B) Pasting temperature, determined by RVA. (C) Enthalpy change for the DSC gelatinisation endotherm. (D) Peak viscosity, determined by RVA. (E) Peak breadth for the DSC gelatinisation endotherm, estimated as difference between the end and onset temperatures. (F) Final viscosity, determined by RVA. Nomenclature of the material is explained in Fig. 1.

The gelatinisation and pasting characteristics of starch from grains of the *sex6* donor parent differed profoundly from those of NFC Tipple and the other donor parents. These characteristics were retained by *sex6* mutant BC_2_F_4_ families but not by the wild-type control. Gelatinisation started at a temperature lower than that for any other line (Fig. 3A). Pasting temperature (Fig. 3B) could not be assessed because of its ill-defined onset. Enthalpy change and peak viscosity (Fig. 3C, D) were also lower than for any other line.

Starch from the *wax* donor parent also had markedly different gelatinisation characteristics from NFC Tipple starch, including higher peak viscosity (Fig. 3D) and lower final viscosity (Fig. 3F). Most of these properties were retained in the *wax* mutant BC_2_F_4_ families but not by the wild-type control.

Starch from the *isa1* donor parent had a lower enthalpy change and peak viscosity than NFC Tipple starch, but neither property persisted in the isa1 mutant BC_2_F_4_ families or the wild-type control (Fig. 3C, D). Properties of starch from the *lys5* donor parent were generally similar to those of NFC Tipple (Fig. 3).

### 3.4 Swelling power and freeze-thaw stability of wax starch

**Fig. 4:**
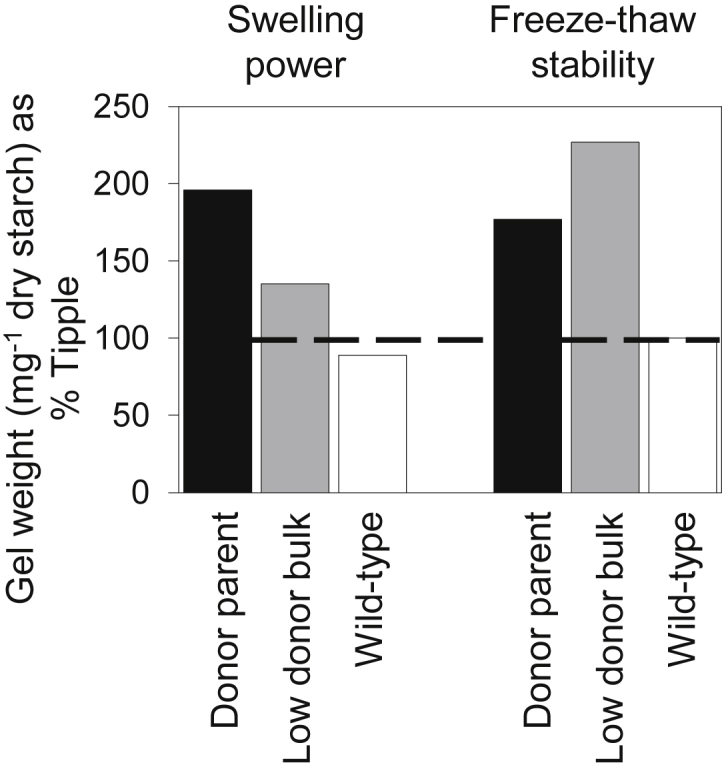
Swelling power and freeze-thaw stability of starches from grain of the *wax* donor parent and *wax* BC_2_F_4_ families. Measurements are expressed as a percentage of the value for NFC Tipple (100%, dashed line). Nomenclature of the material is explained in Fig. 1.

As expected, starch from grain of the *wax* donor parent had higher swelling power and freeze-thaw stability than that of NFC Tipple (Fig. 4). For mutant BC_2_F_4_ families, starch swelling power was lower than in the donor parent but still higher than in NFC Tipple. The freeze-thaw stability of the starch from mutant BC_2_F_4_ families was even higher than for the donor parent.

## 4 Discussion

Comparison of wild-type control and NFC Tipple starch and grain characteristics with those of the mutant donor and mutant BC_2_F_4_ families reveals that some characteristics are very strongly influenced by genetic background, whereas others are largely or solely conferred by the four genes studied.

### 4.1 Grain characteristics and starch content

The *lys5* and *sex6* mutations conditioned low grain weight and low starch content regardless of genetic background. BC_2_F_4_ families carrying either mutation also had low grain weight and starch content regardless of the genomic contribution of the donor parent. This result is consistent with the established importance of the proteins encoded by the *lys5* and *sex6* genes. The ADPglucose transporter of the plastid envelope (encoded by *lys5*) is essential for normal rates of starch synthesis not only in barley but also in maize endosperm, where it is encoded by *Brittle1* (Shannon et al., 1998). Starch synthase IIa (encoded by *sex6*) is also essential for normal rates of starch synthesis in maize and wheat endosperm (Konik-Rose et al., 2007). Barley lines carrying these mutations are thus likely to be intrinsically low yielding, regardless of genetic background.

Introgression of the *isa1* mutation into an NFC Tipple background showed that the low grain weight and starch content of the donor is partly due to genetic background, and probably partly due to *isa1* itself. Mutant BC_2_F_4_ families had higher grain weight and starch content than the donor parent, but values were lower than those of wild-type controls and NFC Tipple even for the BC_2_F_4_ family with the lowest genomic contribution of the donor parent. Because mutant BC_2_F_4_ families had phytoglycogen contents per g flour similar to those of the donor parent but higher starch contents than the donor parent, introgression of the *isa1* mutation into NFC Tipple was accompanied by an increase in the starch to phytoglycogen ratio.

The starch to phytoglycogen ratio in *isa1* mutants of maize [*sugary1* (*su1*) mutants] is also strongly influenced by genetic background. In three rounds of divergent recurrent selection for high starch or high phytoglycogen in a maize population homozygous for a *su1* mutation, Tracey and Chang (2007) were able to generate lines with starch to phytoglycogen ratios ranging from 1.0 to 2.1. As in our study, high starch to phytoglycogen ratios were strongly correlated with high seed weight. Taken together, these results suggest that relatively high grain weight and α-glucan contents can be achieved in barley carrying the *isa1* mutation, but such material will have high starch to phytoglycogen ratios.

As expected from previous studies of *wax* barley, the *wax* mutation had little consequence for starch content (e.g. Oscarsson et al., 1997): the donor parent and BC_2_F_4_ families had starch contents generally comparable with those of NFC Tipple. *Wax* mutants of other cereals also have essentially normal starch contents (e.g. wheat, Graybosch et al., 2003; maize, Creech, 1965). The *wax* donor parent had smaller grains than NFC Tipple, but grain weight of BC_2_F_4_ families was generally similar to those of NFC Tipple.

### 4.2 β-glucan content

Grain of all of the donor parents had higher β-glucan contents (percentage of flour) than that of NFC Tipple. This difference was not simply due to the lower starch content of three donor parents, because introgression of the mutations into NFC Tipple affected the relationship between starch and β-glucan differently in each case. In agreement with these results, previous studies reported high β-glucan contents in grains of the *lys5* donor parent Risø13 (Munck et al., 2004: 2.8 times the content of the wild-type parent Bomi) and in the *sex6* mutant Himalaya 292 (Topping et al., 2003: twice as high as standard barley). However, grain of BC_2_F_4_ families carrying the *lys5*, *isa1* or *sex6* mutations all had lower β-glucan contents than that of the donor parents, even though their starch contents were very differently affected [ranging from the same as the donor parent (*lys5*) to twice as high (*isa1*)]. In the case of *isa1* and *sex6* mutant families, grain β-glucan contents were similar to those of NFC Tipple. It seems unlikely that either gene plays a major role in determining β-glucan content. Nonetheless, all families carrying the *sex6* mutation had higher ratios of β-glucan to starch than NFC Tipple. The β-glucan content was 13% of the starch content for the low-donor family and 6% of the starch content for NFC Tipple. In contrast, the low-donor BC_2_F_4_ family carrying the *lys5* mutation had three times more β-glucan than NFC Tipple. It also had more than twice as much β-glucan as the *sex6* BC_2_F_4_ family, despite that fact that the two families had comparable starch contents.

Although the *wax* donor parent had a relatively modest elevation in β-glucan content in relation to NFC Tipple, this effect was maintained in the mutant BC_2_F_4_ family. This observation suggests that *wax* itself conditions elevated β-glucan content. Correlations between *wax* mutations and high β-glucan contents have been widely observed in barley, in many different backgrounds and for different *wax* mutant alleles (e.g. Baik and Ullrich, 2008; Munck et al., 2004; Ullrich et al., 1986). The reasons for this correlation are not known. Because *wax* mutations do not alter total starch content, the elevated β-glucan content cannot be due to diversion of carbon from amylose synthesis to β-glucan synthesis.

### 4.3 Starch properties

The properties of starch from grain of the *sex6* donor parent and BC_2_F_4_ families carrying this mutation were radically different from those of NFC Tipple and the other donor parents and BC_2_F_4_ families. The starch had very low crystallinity (indicated by the low enthalpy change on gelatinisation) and high heterogeneity (indicated by the wide gelatinisation peak). These properties are consistent with those reported for *sex6* mutants in a different genetic background (Himalaya: Morell et al., 2003). They reflect the radically altered chain length distribution of the amylopectin fraction of starch, the high apparent amylose content and heterogeneous and distorted nature of starch granules from *sex6* mutants (Morell et al., 2003). Loss of the SSII isoform of starch synthase also has a very strong influence on amylopectin structure and hence on granule structure in wheat (Yamamori et al., 2000) and maize (Zhang et al., 2004) endosperm.

Although the properties of starch from the *isa1* donor parent were distinctly different from those of NFC Tipple, at least some of the differences were effects of genetic background. For example, starch from the donor parent had low crystallinity and low peak viscosity relative to NFC Tipple, whereas the mutant BC_2_F_4_ family resembled NFC Tipple in these respects. This background effect may relate to the higher ratio of starch to phytoglycogen in the BC_2_F_4_ family than in the donor parent. *isa1* mutant grain contains a wide range of α-glucan structures, ranging from soluble phytoglycogen to various sizes and shapes of granules and more amorphous material (Burton et al., 2002). It seems likely that insoluble α-glucan extracted from grain of the *isa1* donor parent contains both starch granules and more amorphous α-glucan material with distinct properties. The insoluble α-glucan from the *isa1* BC_2_F_4_ family is likely to have a higher proportion of starch granules than that from the donor parent, and hence properties more like those of starch from NFC Tipple.

Examination of starch from the *lys5* donor parent and BC_2_F_4_ families provided no evidence for a strong influence of *lys5* on starch properties. This observation is consistent with the fact that the *lys5* mutation affects the supply of substrate for starch synthesis in the endosperm (Patron et al., 2002), whereas the other three mutations directly affect the synthesis of the starch polymers.

Starch from BC_2_F_4_ families carrying the *wax* mutation retained the properties generally associated with starches from amylose-free mutants of crop plants. Compared to NFC Tipple, the starch had high swelling power and freeze-thaw stability, high peak viscosity and low final viscosity. However, these parameters were affected to some extent by genetic background. For example, starch from mutant BC_2_F_4_ families had lower swelling power than that of the donor parent, but enhanced freeze-thaw stability. Both of these properties are modulated by the fine structure of amylopectin, in particular the fraction of short glucose chains, and by numerous other factors including phosphate ester content and granule size (Srichuwong et al., 2005, 2012). Further analysis will therefore be required to determine which features of the genetic background contribute to the changes in starch properties that accompany introgression of the *wax* mutation into the NFC Tipple background.

## 5 Conclusions

Introgression of mutations affecting enzymes of starch synthesis into an elite variety by backcrossing has allowed the effects of the alleles to be assessed in a unified genetic background, revealing which starch and grain properties are directly conditioned by the mutations themselves, and which are modulated by genetic background. The *lys5f* allele used in this study conditions exceptionally high contents of β-glucan, much higher than those of *wax* mutants from which barley β-glucan is usually obtained commercially. However, unlike the *wax* mutation, this mutation conditions intrinsically low grain weight. Other mutant alleles of *lys5* (see Patron et al., 2002) might offer more favourable combinations of grain weight and β-glucan content. Although further breeding to enhance grain weight in *isa1* mutant lines is likely to result in higher starch to phytoglycogen ratios, the presence of some phytoglycogen may have advantages for alcohol production and for some food applications. As reported previously, the *sex6* mutation strongly affects starch properties, resulting in a resistant starch with potential health benefits. However, grain weight and starch contents of lines carrying the *sex6* mutation are likely to be intrinsically low. The valuable starch properties of lines carrying the *wax* mutation are modified by genetic background. It seems likely that further breeding could enhance freeze-thaw stability in particular.

## Figures and Tables

**Table 1:** Provenance of lines used in this study.

Material^a^	Genotype	Description	Heterozygosity^b^
NFC Tipple		Recurrent parent	n/a
Risø13	*lys5*	Donor parent	n/a
20-B2-R	*lys5*	High donor line	38.3 (8)
18-A3-R	*lys5*	Medium donor line	18.1 (5)
18-B3-R	*lys5*	Low donor line	10.8 (2)
18-B4-T	*Lys5* (wild-type)	Wild-type control^d^	10.8 (2)
18-B bulk^c^	*lys5*	Low donor bulk	10.8 (2)
Yon M Kei	*wax*	Donor parent	n/a
5-E3-Y	*wax*	High donor line	19.3 (9)
3-C1-Y	*wax*	Medium donor line	11.5 (5)
5-C3-Y	*wax*	Low donor line	7.5 (5)
5-C6-T	*Wax* (wild-type)	Wild-type control^d^	7.5 (5)
5-C bulk^c^	*wax*	Low donor bulk	7.5 (5)
Notch2	*isa1*	Donor parent	n/a
9-C1-N	*isa1*	High donor line	34.0 (9)
8-C2-N	*isa1*	Medium donor line	18.1 (6)
8-B1-N	*isa1*	Low donor line	4.3 (3)
8-B3-T	*Isa1* (wild-type)	Wild-type control^d^	4.3 (3)
8-B bulk^c^	*isa1*	Low donor bulk	4.3 (3)
Sex6	*sex6*	Donor parent	n/a
14-A2-X	*sex6*	High donor line	23.4 (7)
11-B4-X	*sex6*	Medium donor line	17.2 (2)
14-H3-X	*sex6*	Low donor line	8.2 (3)
14-H1-T	*Sex6* (wild-type)	Wild-type control^d^	8.2 (3)
14-H bulk^c^	*sex6*	Low donor bulk	8.2 (3)

a Codes refer to material developed by Howell et al. (2014).

b Percentage of the BC_2_ progenitor genome which remained heterozygous, estimated from BC_2_ genotypes and the barley DArT consensus map (Wenzl et al., 2006); the number of distinct BC_2_ heterozygous segments is shown in parentheses.

c Bulks were made by pooling equal quantities of grain from low donor lines as follows. *lys5*: lines 18-A1-R, 18-A2-R and 18-A3-R. *wax*: lines 5-C1-Y to 5-C5-Y. *isa1*: lines 8-B1-N and 8-B2-N. *sex6*: lines 14-H2-X and 14-H3-X.

d For each mutation, the wild-type control and the low donor line represent a pair of full-sib near-isogenic lines, selected as contrasting BC_2_F_2_ homozygotes from within a segregating family.
