# Supplementary material for: Use of advanced recombinant lines to study the impact and potential of mutations affecting starch synthesis in barley
Source: J Cereal Sci. 2014 Mar;59(2):196–202. doi: 10.1016/j.jcs.2013.12.012 (PMC3990431; doi:10.1016/j.jcs.2013.12.012)
Supplement: Supplementary file 1 [file mmc1.docx]

**Use of advanced recombinant lines to study the impact and potential of mutations affecting starch synthesis in barley**

Thomas P. Howard, Brendan Fahy, Fiona Leigh, Phil Howell, Wayne Powell, Andy Greenland, Kay Trafford, Alison M. Smith

**Supplementary data**

**Supplementary Table 1. Grain weight, width, length and area.**

Values are those from which Fig. 1 was derived. Values are means of measurements on between 230 and 370 grains, ± SEM. Nomenclature of the material is explained in Fig. 1. TGW is thousand-grain weight.

|  | TGW(g) | Area (mm^2^) | Width (mm) | Min Width | Max Width | Length (mm) | Min Length | | Max Length |
| --- | --- | --- | --- | --- | --- | --- | --- | --- | --- |
| NFC Tipple | 57.51 | 31.0 ± 0.3 | 4.3 ± 0.03 | 3.1 | 5.6 | 9.7 ± 0.07 | 5.5 | 11.4 | |
| *lys5* |  |  |  |  |  |  |  |  | |
| Donor parent | 43.29 | 30.4 ± 0.6 | 3.8 ± 0.04 | 2.5 | 5.1 | 10.4 ± 0.13 | 6.3 | 14.4 | |
| High donor | 38.15 | 30.6 | 3.9 | 2.7 | 5 | 10.1 | 7.5 | 13.6 | |
| Medium donor | 34.63 | 30.0 | 3.8 | 2.3 | 4.9 | 10.1 | 6.7 | 16.7 | |
| Low donor | 36.77 | 30.7 | 3.9 | 2.7 | 4.8 | 10.2 | 7.1 | 13.1 | |
| Low donor bulk | 37.13 | 30.5 ± 0.3 | 3.9 ± 0.03 | 2.7 | 5.3 | 10.3 ± 0.07 | 7.6 | 12.3 | |
| Wild-type | 54.14 | 30.4 ± 0.3 | 4.2 ± 0.04 | 2.7 | 5.3 | 9.6 ± 0.06 | 5.9 | 13 | |
| *wax* |  |  |  |  |  |  |  |  | |
| Donor parent | 41.63 | 22.1 ± 0.2 | 3.7 ± 0.02 | 2.5 | 4.6 | 7.7 ± 0.06 | 4.5 | 9.9 | |
| High donor | 51.59 | 29.5 | 4.0 | 2.4 | 5.8 | 9.8 | 6.8 | 15.1 | |
| Medium donor | 51.85 | 25.4 | 4.1 | 2.8 | 4.9 | 8.1 | 6.3 | 11.8 | |
| Low donor | 55.06 | 30.0 | 4.1 | 2.8 | 5.8 | 9.6 | 6.7 | 12.2 | |
| Low donor bulk | 53.08 | 28.8 ± 0.4 | 4.1 ± 0.04 | 3 | 6.2 | 9.4 ± 0.09 | 5.4 | 11.4 | |
| Wild-type | 56.25 | 29.2 ± 0.6 | 4.2 ± 0.04 | 2.9 | 5 | 9.4 ± 0.11 | 5.1 | 11.6 | |
| *isa1* |  |  |  |  |  |  |  |  | |
| Donor parent | 30.24 | 28.9 ± 0.8 | 3.5 ± 0.06 | 1.7 | 7.1 | 11.0 ± 0.20 | 4.9 | 18.6 | |
| High donor | 40.34 | 30.7 | 4.0 | 2.6 | 5.1 | 9.8 | 5.9 | 14.4 | |
| Medium donor | 47.14 | 31.4 | 4.2 | 3.2 | 5.2 | 9.9 | 6.7 | 14.7 | |
| Low donor | 47.68 | 30.5 | 4.1 | 2.4 | 5.1 | 9.7 | 6.5 | 12.2 | |
| Low donor bulk | 47.05 | 30.8 ± 0.5 | 4.2 ± 0.05 | 2.8 | 5.3 | 9.8 ± 0.10 | 5.2 | 14.8 | |
| Wild type | 57.78 | 31.3 ± 0.4 | 4.3 ± 0.04 | 2.7 | 5.3 | 9.6 ± 0.09 | 6.6 | 12 | |
| *sex6* |  |  |  |  |  |  |  |  | |
| Donor parent | 35.3 | 34.4 ± 0.7 | 4.0 ± 0.07 | 2.6 | 6 | 11.3 ± 0.13 | 8.4 | 17.5 | |
| High donor | 37.45 | 33.5 | 4.3 | 3 | 5.5 | 10.2 | 5.7 | 12.9 | |
| Medium donor | 34.51 | 30.1 | 4.0 | 2.9 | 5.8 | 9.9 | 7 | 14 | |
| Low donor | 37.51 | 30.1 ± 0.5 | 3.9 ± 0.04 | 2.5 | 6.1 | 9.9 ± 0.13 | 5.4 | 17 | |
| Wild type | 55.48 | 30.2 ± 0.4 | 4.2 ± 0.05 | 2.4 | 5.6 | 9.6 ± 0.08 | 6.2 | 13.8 | |

**Supplementary Table 2. Starch contents of flour.**

Values are those from which Fig. 2A was derived. They are means ± SEM of measurements on three samples of flour. Nomenclature of the material is explained in Fig. 1. Moisture contents (% weight) of flours of mutant donor, low-donor bulk and wild-type controls were, respectively: *lys5* 8.0, 12.9, 7.9; *wax* 9.1, 13.0, 11.6; *isa1* 7.1, 10.9, 12.0; *sex6* 6.8, 8.9, 11.7. The moisture content of Tipple flour was 10.6%.

| Plant material | Starch content (mg g^-1^ flour) |
| --- | --- |
| NFC Tipple | 539. 5 ± 8.4 |
|  |  |
| *lys5* donor parent | 229. 2 ± 4.0 |
| *lys5* high donor | 223.1 ± 1.0 |
| *lys5* medium donor | 267.8 ± 7.4 |
| *lys5* low donor | 225.6 ± 4.7 |
| *lys5* low donor bulk | 226.1 ± 4.1 |
| *Lys5* wild type control | 514.8 ± 18.2 |
|  |  |
| *wax* donor parent | 501.3 ± 4.6 |
| *wax* high donor | 485.2 ± 7.6 |
| *wax* medium donor | 518.1 ± 8.7 |
| *wax* low donor | 444.6 ± 7.7 |
| *wax* low donor bulk | 471.0 ± 5.5 |
| *Wax* wild type control | 524.9 ± 8.7 |
|  |  |
| *isa1* donor parent | 225.3 ± 6.5 |
| *isa1* high donor | 296.2 ± 8.3 |
| *isa1* medium donor | 355.2 ± 9.1 |
| *isa1* low donor | 383.6 ± 8.5 |
| *isa1* low donor bulk | 379.1 ± 3.9 |
| *Isa1* wild type control | 520.6 ± 12.5 |
|  |  |
| *sex6* donor parent | 173.5 ± 2.5 |
| *sex6* high donor | 283.7 ± 13.2 |
| *sex6* medium donor | 249.7 ± 11.2 |
| *sex6* low donor | 273.0 ± 8.4 |
| *Sex6* wild type control | 520.3 ± 7.4 |

**Supplementary Table 3. Starch and soluble glucan contents in flour from grain of NFC Tipple, the *isa1* donor parent and *isa1* BC_2_F_4_ families.**

Values are those from which Fig. 2B was derived. They are means ± SEM of measurements on three samples of flour. Measurements were on different samples of grain from those shown in Fig. 2A. Nomenclature of the material is explained in Fig. 1.

|  | Starch content (mg g^-1^ flour) | Soluble glucan content (mg g^-1^ flour) |
| --- | --- | --- |
| NFC Tipple | 555 ± 31 | 71 ± 4 |
|  |  |  |
| Donor parent | 219 ± 11 | 124 ± 19 |
|  |  |  |
| High donor | 262 ± 5 | 118 ± 5 |
|  |  |  |
| Medium donor | 307 ± 5 | 108 ± 3 |
|  |  |  |
| Low donor | 352 ± 20 | 116 ± 34 |
|  |  |  |
| Low donor bulk | 343 ± 30 | 94 ± 11 |
|  |  |  |
| Wild-type | 603 ± 77 | 74 ± 6 |
|  |  |  |
